# Combining ethnography and conversation analysis to explore interaction in dementia care settings

**DOI:** 10.1111/hex.13563

**Published:** 2022-07-16

**Authors:** John Chatwin, Katherine Ludwin, Isabelle Latham

**Affiliations:** ^1^ Research and Innovation Midlands Partnership NHS Foundation Trust Stafford UK; ^2^ Learning & Development Hallmark Care Homes Essex UK

**Keywords:** conversation analysis, dementia care, ethnography, video‐ethnography

## Abstract

**Background:**

It is well established that the actions and behaviour of care home workers are fundamental to the well‐being of the people they care for. They not only deliver basic care but through their day‐to‐day presence provide an underlying continuity for residents, many of whom will have dementia or other cognitive problems. This can have many positive psychological and social benefits. A variety of ethnographic approaches have been used to explore the broader social and cultural dimensions of dementia care work. Similarly, there is a growing body of work applying micro‐level approaches such as conversation analysis (CA) to describe the interactional mechanics of specific care skills.

**Strategy:**

We outline what ethnography and CA are, how they work as stand‐alone methodologies and how they have been used in care work and dementia care settings. A working illustration is given of how the two approaches may be integrated.

**Discussion:**

Dementia care workers occupy a uniquely tenuous sociopolitical and professional position within healthcare. If they are to progress to a more professional status there is a pressing need for standardized systems of training to be developed. As has been common practice in most other fully professionalized sectors of healthcare, this training needs to be backed up by an understanding of how effective care work is undertaken at the micro‐level. For it to be practically relevant to care workers it also needs to have been informed by the wider social context in which it occurs.

**Conclusion:**

We argue that elements of ethnography and CA can be usefully combined to provide the fully contextualized micro‐level descriptions of care work practice that will be needed if current moves towards the greater professionalization of care work are to continue.

**Patient or Public Contribution:**

The authors undertake a significant amount of Patient and Public Involvement and Engagement and study codesign with members of the public, care workers and people living with dementia. Our engagement work with care staff and family carers undertaken as part of a current National Institute for Health Research study exploring naturalistic care worker skills (see acknowledgements) has been particularly relevant in shaping this article.

## 1 INTRODUCTION

Recent years have seen major policy initiatives in the United Kingdom (UK) aimed at improving residential and dementia‐related care at both medical and psychosocial levels.^1^, ^2^ Significantly, emphasis has been placed on research, which addresses day‐to‐day issues for people in long‐term care.^3^, ^4^ By definition, frontline care staff are centrally implicated in this process and understanding their role and activities needs to be high on the research agenda.^5^, ^6^ However, despite their widely acknowledged importance care home workers continue to be a marginalized sector of the healthcare community.^3^, ^7^, ^8^ While care settings are regulated, in the UK, by the Care Quality Commission, there is no guiding or regulating body for frontline carers themselves. They face one of the most challenging and at times demoralizing roles yet they are largely undertrained, low paid and undervalued.^3^ Although care work routinely involves nursing‐related tasks it has historically been perceived as demeaning and very much a ‘last resort’ career choice, particularly by professionals from a nursing background.^7^ Similarly, a significant number of care home staff are nurses who are unable to formally practice as in the UK because their qualifications may have been gained in other countries and are not automatically recognized here. As yet care workers in the UK have limited career pathway choices,^9^, ^10^ and in society in general, perceptions about the motivation and commitment of people who work in the sector are traditionally negative.^11^ Their already vague professional status is continually eroded by media coverage, which all‐too‐often focuses on extreme cases of poor practice.^12^, ^13^ Even the significant rise in public awareness of care work that has resulted from the COVID‐19 pandemic^14^ may only have had a limited impact. Without significant intervention, entrenched systemic limitations around pay, workforce training and uncertain career trajectories seem likely to continue.

Dementia care workers occupy a uniquely tenuous sociopolitical and professional position within healthcare. Progression to more professional status for this group requires, in part, the development of standardized systems of training. As has been common practice in most other fully professionalized sectors of healthcare, this training needs to be backed up by an understanding of how effective care work is undertaken at the micro‐level. For it to be practically relevant to care workers it also needs to have been informed by the wider social context in which it occurs. In this article, we focus on two distinct sociological methodologies—*Ethnography* and *Conversation Analysis (CA)*—that have already been used to offer detailed meso and micro perspectives on dementia care and the activities of dementia care workers. In research terms, meso is concerned with interactions within or between groups to develop an understanding of context and culture whereas micro focuses on small‐scale interactions between individuals. We argue that elements of both approaches can be usefully combined to provide the fully contextualized micro‐level descriptions of care worker practice that will be needed if current moves towards greater professionalization are to continue.

## 2 ETHNOGRAPHY

Ethnography is a wide and varied discipline, encompassing an eclectic range of modalities.^15^, ^16^ It has its roots in colonialist anthropology that sought to study ‘primitive’ cultures outside of Europe during the early 1800s,^17^ but is now more often associated with exploring the experiences of socially marginalized communities and subcultures.^18^, ^19^, ^20^, ^21^ The classic ethnographic method is participant observation—often with a researcher spending months or years actively involved with the group they are studying.^22^ It is also often the case that the communities and subcultures under investigation are ones that the researcher is intimately familiar with.^23^ Along with participant observation, ethnographers may employ a variety of other (primarily qualitative) methods. These might include in‐depth qualitative interviews; the collection and analysis of documents; the analysis of participant photographs and written accounts; engaging participants in the creation of audio and video diaries and the use of naturalistic film as a primary data source. Many modern‐day Ethnographies in health and social care have moved away from the classic ‘deep‐emersion’ model,^24^ and it is not uncommon to find ethnographic work that uses short‐term engagement in the field, with researchers visiting intermittently or for short periods of time.^25^

### 2.1 Ethnography in dementia care settings

Ethnography is a highly flexible methodology, in which types, locations and focus of data collection are often determined by developments in the field and the emerging understanding of the researcher.^23^ As such, it has become an approach commonly used to examine dementia care settings. Bourbonnais and Ducharme,^26^ for example, engaged with people living with dementia, care staff, and family members to explore their perceptions of meaning in relation to screams, while Stephens et al.^24^ used ethnography to explore the ways in which transitional objects are used by people living with dementia. Ethnographic studies in dementia care environments have focused on aspects of independence and daily living^27^; levels of care home resident engagement^28^; activity and communication^29^, ^30^ and person‐centred care.^31^ Studies have also addressed the intricate relational dynamics that develop between family carers, care staff and the person being cared for, and have highlighted problematic areas, such as the disparity between the socially‐oriented interests of residents and the task‐based agenda of the care staff.^32^, ^33^

Along with the extensive body of general care‐environment research, there is a significant subset of work using Ethnography to focus on the role of care staff and issues around their perceptions of self and professional identity. Systemic issues, such as the disproportionate number of women in care work and the patterns of inequality that have resulted from this have also been addressed.^34^, ^35^ Building on early work by Lanceley^36^ and Coupland et al.,^37^ which explored how caring encounters were influenced by wider conditions in care homes, a study by Hubbard et al.^38^ also highlighted how the location and context of interaction between care staff and residents impacts on quality of care. Work more specifically concerned with the lived experience of care workers themselves, rather than their direct or indirect influence on the people they care for is also now gaining popularity. Bailey et al.,^39^ for example, focused on the way in which hospital inpatient dementia care workers cope with the emotional demands of their role, and in their ethnography of care work in three National Health Service dementia wards, Scales et al.^40^ described how efforts to encourage care staff to provide person‐centred care were undermined by an underlying sense of powerlessness. Similar negative undercurrents were uncovered in an ethnography of hospital dementia care workers conducted by Lloyd et al.^41^ In this case, however, it was the development of a strong group identity among the care workers who were the subject of the study that was problematic. The emergence of a strong ‘us’ and ‘them’ attitude created significant barriers to the development of effective teamworking with other groups of healthcare professionals.

## 3 CONVERSATION ANALYSIS

In strong contrast to ethnography, CA is a methodology that, in its classic form at least, focuses on delivering a detailed description of the micro‐level ‘rules’ that underly human interaction^42^; rules that are largely independent of setting or context. CA originates in sociology but draws on insights from other disciplines, such as psychology and linguistics.^43^ Utilizing video and audio recordings of *naturally occurring* interaction, and a highly detailed method of transcription that aims to capture the minutiae of speech and aspects of nonverbal behaviour (see Box 1), CA aims to study the structure and order of naturally occurring talk in interaction. It is an approach that has been widely used to investigate healthcare and medical settings. It has, for example, been applied to primary care interactions,^44^ health visiting,^45^ counselling,^46^ mental health,^47^ specialist neurological consultations^48^ and a variety of complementary and alternative medicine settings.^49^ Many studies have been concerned with providing a broad sociolinguistic analysis of the features of particular environments but work has also focused on exploring specific aspects of interaction within these settings. Such as the ways in which patients ‘frame’ their presenting complaints^50^; how health professionals offer diagnostic information to patients^51^ and how various types of health professionals interact differently with the people they care for.^52^

Box 1 **Simplified CA transcription symbols**

In CA, punctuation symbols such as full stops, commas and question marks etc., are used to denote the characteristics of ongoing speech and do not necessarily maintain a conventional grammatical function. The example in this article have been simplified for clarity, but the meanings of the symbols that have been used are:

.—Full stops are used to indicating a falling intonation.

,—Commas indicate continuing intonation.

CAP—Capital letters indicate speech that is louder compared to the surrounding talk.

Under—Underlined speech indicates emphasis on a word or part of a word.

(0.5)—Numbers within brackets indicate timings in whole and tenths of a second.

(.)—A full stop within brackets indicates a ‘micro pause' of less than two tenths of a second.

{—Comments and observations are written between {Indented brackets}

((—Double brackets indicate unclear or difficult to hear speech.

[—Square brackets are used to denote overlapping speech, so if, as is common in conversational speech, one person anticipates how the other's turn will end and begins their turn before it is fully complete, the transcript looks like this:

30    Tom:    That's it (0.5) ay[e

31    Sarah:                    [Yea? (0.5) How's that?

### 3.1 CA in dementia care settings

CA‐based research on aspects of interaction involving people living with dementia is fairly well represented. At a therapeutic level, for example, work by Sommerbeck^53^ offered insights into the interactional difficulties of engaging people with severe dementia in psychotherapeutic processes. She outlined a ‘pre‐expressive’ approach, which attempted to incorporate literal reflections of the client's verbal as well as nonverbal behaviour. Topic management in dementia communication was the focus of work by Hall et al.,^54^ and the idiosyncratic features of laughter as an interactional resource by people living with dementia have been the subject of work by Wilson et al.^55^ There have also been attempts to use CA as a means of developing interactionally based interventions for the screening and diagnosis of dementia. Elsey et al.^56^ and Jones et al.,^57^ for example, explored whether the profile of a patient's verbal and nonverbal interaction with a doctor in memory clinic sessions could help differentiate between functional memory disorders and memory problems related to dementia.

As yet, there has been limited CA research specifically focusing on dementia care workers, with only a handful of studies adopting this specific angle. Notable exceptions being early work by Cohen‐Mansfield and Werner^58^ who examined the management of verbally disruptive behaviours in nursing homes, and Åkerström^59^ who described the way in which dementia‐care workers formulated their talk about aggressive patients. More recently, Allwood et al.^60^ utilized CA to describe how healthcare professionals in hospital care settings drew their interactions with patients to a close. Recent studies by Jones,^61^ and Young et al.,^62^ also explored issues such as problematic communication patterns between people living with dementia and people they frequently engage with (e.g., care staff or family carers).

## 4 INTEGRATION

It is evident that although ethnography and CA offer very different standpoints (i.e., meso and micro) they share an important affinity.^63^ CA is largely concerned with how sequences of (primarily verbal) interaction perform social action and involves capturing audio and video recordings of naturalistic behaviour. Ethnography has traditionally relied on participant observation and interview to capture naturalistic interaction, with emergent subdisciplines, such as video‐ethnography^64^ and participatory video,^65^ often providing data that is highly compatible with the CA approach. At another level, the detailed analysis of recordings and transcripts that is a feature of CA could be regarded as a form of intense ethnographic observation.^66^ Although CA has traditionally been concerned purely with the decontextualized mechanisms of interaction,^67^ the emergence of ‘applied’ CA^68^ has connected the discipline more closely with the broader socio‐political issues that motivate much ethnographic work.

We suggest that in settings such as the dementia care arena that we are concerned with, and depending on what a given study is seeking to uncover, there is much to be gained from combining elements of both methodologies.

To show how useful connections between CA and ethnography can be achieved at a practical level, we will focus on an example taken from a study that is currently utilizing a combination of video‐ethnography and CA to explore aspects of dementia care worker interaction.^69^ In the study, a video‐ethnographic approach is being used to record naturalistic activity in a variety of dementia care settings, with the underlying aim of capturing instances of care workers using ‘natural’ therapeutic skills. That is, skills or ways of behaving that they have not necessarily had any formal training in, but which appear to have a positive impact on the wellbeing of the people they care for. CA is then being used to establish how these encounters work at the micro‐level. In this case, the CA and video‐ethnographic elements of the study essentially produce their own discrete datasets, with neither approach taking priority. For the sake of clarity, however, here we use a piece of self‐contained CA data as our starting point. Extract 1 (below) is a short CA format transcription^70^ of an encounter filmed at a secure dementia day‐care centre that took part in the study. It involves Tom, an elderly man attending the centre, and Sarah, one of the care workers looking after him. (For an explanation of the transcription system used to present CA data, see Box 1). Tom is living with dementia and has limited awareness of where he is. He has become restless and wants to leave. The extract opens as he approaches one of the secure doors to the day centre lounge and is intercepted by Sarah:

Extract 1


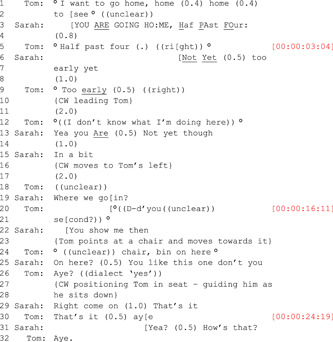


Taking the CA transcript in isolation, the extract can be seen as a discrete sequence of turns at talk, each one produced in response to the turn preceding it and leading to a particular outcome. Using the established CA method, the extract would usually form part of a large corpus of similar, self‐contained examples (large‐scale studies might work with 200 or more examples). These would then be analysed to isolate reoccurring features, such as particular types of turn construction that might result in different types of outcomes. Individual CA transcripts, such as Extract 1, can also be usefully analysed in isolation. When this *applied* approach^68^ is used, the focus can be more context‐relevant and may, as in this case, be concerned with the way in which people who are interacting with one another adapt their turns at a talk within established conversational norms.

From a broad CA reading of Extract 1, several features stand out. It appears that Sarah's initial responses to Tom's turn at the opening of the sequence (Line 1) ‘I want to go home…’ reflects a degree of alignment. Although she speaks loudly, which contrasts with Tom's very quiet delivery (indicated by the superscript ‘O's’) and talks over him well before he has completed his turn (Line 2), she is providing a response which indicates that at this point she is closely attending to what Tom is saying. Later in the sequence, however (starting at Line 10, when Tom says he doesn't know where he is), it appears that Sarah disregards this and continues to orient to his initial request to leave (Line 1). Further on in the sequence too, it appears that she does not directly respond to what Tom actually says, and frequently talks over him (Lines 20 and 23). So, although on one level this encounter might be regarded as being successfully resolved; Sarah has distracted Tom from trying to leave and the situation has not escalated. On another level, the structural and sequential detail reveals that from an interactional perspective, it was perhaps less even‐handed.

### 4.1 The ethnographic perspective

The ethnographic data relevant to this extract was collected over a 2‐month period of participatory observation and naturalistic video recording in and around the day‐care centre. In the tradition of immersive ethnography,^71^ members of the research team spent many hours filming people going about their routine daily activities and engaged in numerous spontaneous, informal interviews. The team also used a *Stimulated Recall (SR)* process, which involved playing back sequences of video to staff to get their perspectives on what they thought was significant; why a given interaction had gone well or not so well and why certain situations were more challenging than others. In a short SR session immediately after the interaction with Tom, Sarah explained that he regularly made requests to go home and she had consciously used a distraction strategy that, from experience, she knew he would be likely to respond to. It also emerged over the course of our time in the centre that the rest of the staff were equally familiar with how best to engage Tom during these situations and essentially saw them as an indication of stress or low mood.

**Figure 1:**
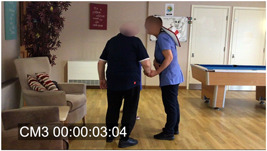
Tom asks about the time (corresponding to Lines 2–5 in Extract 1).

**Figure 2:**
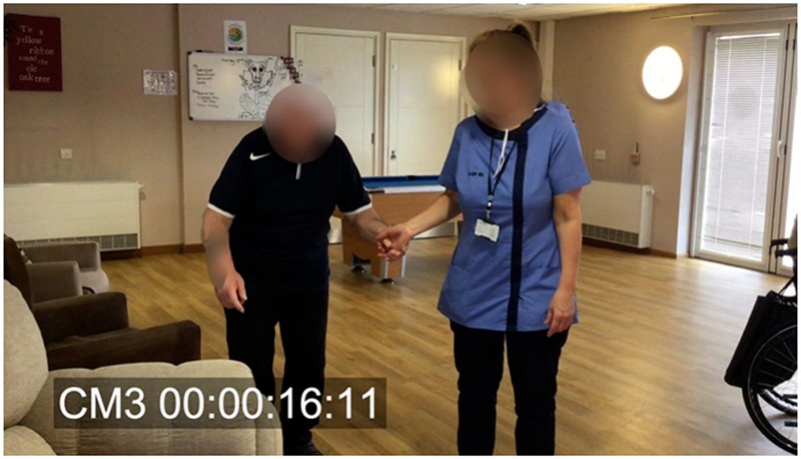
Sarah leads Tom to the chair (corresponding to Lines 19–20 in Extract 1).

**Figure 3:**
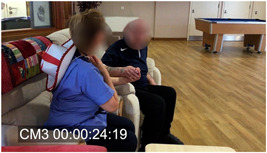
Sarah and Tom sit down (corresponding to Lines 27‐32 in Extract 1).

Other relevant ethnographic details included the fact that Tom was very hard of hearing, had difficulty communicating and walking and tended to mumble very quietly to himself. It was also evident that touch and tactility played a significant role in Sarah's approach to communicating with him. She emphasized how important a confident use of touch could be with people living with dementia as their other channels of communication diminish. Figures 1, 2, 3 are frames taken from the original video of Extract 1 (corresponding time code in square brackets on Extract 1). For the entire encounter, Sarah holds Tom's hand and uses this contact to show reassurance, but also as a key part of her distraction strategy; she leads him away from the door and into the seating area while the verbal elements of the interaction take place—essentially as a simultaneous but distinct layer of interaction. Similarly, the frames illustrate how Sarah holds continuous eye contact as she engages with Tom.

It can be seen that although both the Ethnographic and CA data provide useful self‐contained descriptions of the encounter between Sarah and Tom, there are elements that only become clear when both perspectives are considered. Without the ethnographic details about Sarah's approach to using touch and contact, for example, the CA extract alone might not have captured the significance of the simultaneous layers of verbal and nonverbal behaviour; without the CA perspective, an accurate record of how the encounter unfolded on a turn‐by‐turn basis would not have been available, and neither would significant details about the relationships between these turns, their sequential progression, and how this led to the outcome of the encounter.

Combining ethnography and CA can have other benefits too, particularly when research findings need to be developed into something that is of practical use. In the case of the study that provided our Sarah and Tom interaction, for example, a secondary aim has been to explore the possibility of using original fieldwork recordings as part of a video‐based training resource for dementia care workers. A resource that, because it involves real care workers looking after real people in real care settings, retains a high degree of authenticity.

## 5 CONCLUSIONS AND IMPLICATIONS FOR PRACTICE

The marginalization and underrepresentation, which have traditionally formed the backdrop to dementia care work, have meant that it has been particularly attractive to the sociological investigation. However, in terms of supporting moves towards the greater professionalization of care work, this may have been a disadvantage. Care environments and the people who populate them, either as workers, residents or carers have often been approached as if they were elements of an interesting subculture rather than an integrated part of the healthcare system. This has meant that whereas research focusing on specific aspects of professional behaviour—for example, particular types of clinical interaction or consultation processes—are well‐established in mainstream healthcare research, in care work, it has more often been broader sociopolitical issues that have been of interest.

We have shown that while ethnography and CA offer very different sociological perspectives, they share an affinity which makes them a particularly effective combination for redressing this imbalance. Care work continues to struggle for professional recognition. To move closer to this goal, a key requirement will be the development of standardized and accredited training that can compete on equal terms with other sectors of healthcare such as nursing. If this training is to be effective it needs to be directly relevant to care workers themselves, and grounded in a micro‐level understanding of how care work is actually undertaken.

There can be limitations in conducting studies of the kind we have outlined—such as gaining in‐depth access to dementia care settings and the level of time and commitment studies can demand from care homes and care staff. However, if it can be achieved, combining the meso and micro perspectives of CA and ethnography is an extremely effective way to isolate fundamental features of care work practice. More importantly, it offers a way to understand how these features work at a micro‐level so they can inform training that resonates with the everyday reality of care work.

## CONFLICT OF INTEREST

The authors declare no conflict of interest.

## ETHICS STATEMENT

Ethical approval for the study was granted by the West Midlands—Coventry & Warwickshire Research Ethics Committee NHS ethics committee in November 2020 (15/YH/0184).

## Data Availability

Data sharing is not applicable to this article as it is a review and no new datasets were generated.
